# Abundant *Sulfitobacter* marine bacteria protect *Emiliania huxleyi* algae from pathogenic bacteria

**DOI:** 10.1038/s43705-023-00311-y

**Published:** 2023-09-22

**Authors:** Roni Beiralas, Noy Ozer, Einat Segev

**Affiliations:** Department of Plant and Environmental Sciences, The Weizmann Institute of Science, Rehovot, Israel

**Keywords:** Water microbiology, Symbiosis

## Abstract

*Emiliania huxleyi* is a unicellular micro-alga that forms massive oceanic blooms and plays key roles in global biogeochemical cycles. Mounting studies demonstrate various stimulatory and inhibitory influences that bacteria have on the *E. huxleyi* physiology. To investigate these algal-bacterial interactions, laboratory co-cultures have been established by us and by others. Owing to these co-cultures, various mechanisms of algal-bacterial interactions have been revealed, many involving bacterial pathogenicity towards algae. However, co-cultures represent a significantly simplified system, lacking the complexity of bacterial communities. In order to investigate bacterial pathogenicity within an ecologically relevant context, it becomes imperative to enhance the microbial complexity of co-culture setups. *Phaeobacter inhibens* bacteria are known pathogens that cause the death of *E. huxleyi* algae in laboratory co-culture systems. The bacteria depend on algal exudates for growth, but when algae senesce, bacteria switch to a pathogenic state and induce algal death. Here we investigate whether *P. inhibens* bacteria can induce algal death in the presence of a complex bacterial community. We show that an *E. huxleyi-*associated bacterial community protects the alga from the pathogen, although the pathogen occurs within the community. To study how the bacterial community regulates pathogenicity, we reduced the complex bacterial community to a five-member synthetic community (syncom). The syncom is comprised of a single algal host and five isolated bacterial species, which represent major bacterial groups that are naturally associated with *E. huxleyi*. We discovered that a single bacterial species in the reduced community, *Sulfitobacter pontiacus*, protects the alga from the pathogen. We further found that algal protection from *P. inhibens* pathogenicity is a shared trait among several *Sulfitobacter* species. Algal protection by bacteria might be a common phenomenon with ecological significance, which is overlooked in reduced co-culture systems.

## Introduction

Micro-algae have evolved with bacteria in their surroundings for millions of years [1]. This co-evolution gave rise to various interactions between algae and bacteria that influence the involved microorganisms and their environment [2, 3]. Research on natural bacterial communities that are associated with algae elucidated important aspects of the ecology and dynamics of algal-bacterial communities, and the influence of such communities on microbial physiology. Studies revealed the structure of bacterial communities associated with algae in the environment, the key bacterial members within these communities, and specificity between bacteria and algal hosts [4–7]. Algal derived metabolites were shown to be key in shaping the community structure and in turn, microbial physiology [7–10].

Environmental research was complemented by studies of controlled and reduced laboratory algal-bacterial co-culture systems [10–27]. Such co-cultures allow a detailed understanding of the mechanisms underlying interactions between specific algae and bacteria. The study of algal-bacterial co-cultures revealed that bacteria from the Roseobacter group [28] interact with various algal groups such as dinoflagellates, diatoms and coccolithophores, and that these interactions span from mutualism to pathogenicity [11, 14, 16, 18–27].

Studies on algal-bacterial co-cultures deepened our understanding of specific interactions between algae and bacteria, while environmental research shed light on natural algal-associated bacterial communities. Both approaches have limitations; the environmental approach is restricted in its ability to offer mechanistic insights and the co-culture approach cannot encompass the complexity of the marine environment. While some results that were achieved in the lab align with observations at sea [29], there are instances where laboratory findings contrast with environmental observations. As an example, algal-bacterial co-cultures often revealed pathogenic behavior of Roseobacter bacteria towards their algal hosts [14, 16, 19–24]. Yet many of these Roseobacters were originally isolated from natural communities associated with algae at sea, in which the pathogenic behavior that results in algal death is not observed [30–32]. Therefore, a model system with increased microbial complexity is needed in order to resolve the pathogenic nature of various Roseobacters and its relevance in the environment.

In recent years, the use of synthetic communities was highly effective in the study of complex host-microbiome interactions. Synthetic communities, or in short syncoms, are commonly used in gut and root microbiome research, due to their ability to capture some of the natural complexity yet in a simplified and controlled manner [33]. Syncoms are an intermediate model system between co-cultures and natural communities that enable the study of host-bacteria as well as bacteria-bacteria interactions in the context of a community. Syncoms have allowed the identification of keystone species and functions in the community and unveiled microbiome-mediated phenotypes [34].

*Emiliania huxleyi* is the most abundant coccolithophore species in modern oceans [35]. Different strains of these algae exhibit high genome variability which allows them to thrive in different environmental conditions [36]. During the spring and summer time, populations of *E. huxleyi* form massive annual blooms that can reach cell densities of up to 10^8^ cells/l in temperate and subpolar regions [37]. These micro-algae contribute significantly to the oceanic carbon cycle through both photosynthesis and the formation of calcium carbonate scales [37].

In this study, we isolated bacteria associated with an *E. huxleyi* strain and constructed an algal-bacterial syncom to investigate bacterial behavior, with a focus on the pathogenicity of bacteria within the community. The results showed that the bacterial pathogen *Phaeobacter inhibens* was unable to trigger algal death when it was part of a bacterial consortium. Using the novel syncom, a single bacterial species from the abundant *Sulfitobacter* genus was identified as a protector of the algae from pathogenic *P. inhibens* bacteria. Several other species from the *Sulfitobacter* genus were also found to possess the ability to protect algae from the pathogen.

The bacterial ‘protector’ phenotype might be as common as the wide distribution of algal-associated *Sulfitobacter* species, and therefore, is of ecological significance. These findings highlight the centrality of bacteria-bacteria interactions in shaping host-microbiome dynamics and expand current knowledge on the possible roles of bacteria within a community.

## Results

### The alga *Emiliania huxleyi* harbors a bacterial community that includes members of the Roseobacter group

**Fig. 1:**
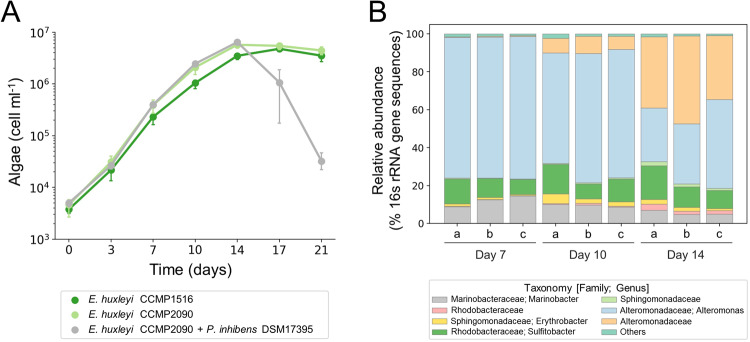
Algae grow with a bacterial community of marine bacteria. **A** Growth of the non-axenic *E. huxleyi* CCMP1516, axenic *E. huxleyi* CCMP2090 and *E. huxleyi* CCMP2090 with the bacterium *P. inhibens* DSM17395 was monitored for 21 days. As can be seen, the presence of the bacterium *P. inhibens* DSM17395 triggered algal death, while the bacterial community that is associated with the algae (in strain *E. huxleyi* CCMP1516) did not impact algal growth. Each data point consists of 3 biological replicates, error bars designate ± SD. **B** The different bacterial taxa associated with *E. huxleyi* CCMP1516. Relative abundance was calculated as percentage of 16S rRNA gene sequences along three time points of algal growth. Each time point consists of 3 biological replicates (a, b and c). Bacteria were classified to the genus or family level. Two taxa with a relative abundance lower than 1% across all samples were pooled together and designated as “Others”. These taxonomies were classified as belonging to the *Piscirickettsiaceae* family and the Alteromonadales order.

Often, bacterial pathogenicity is observed when the alga *E. huxleyi* is co-cultured with a single bacterial species that is naturally found in the algal microbiome of environmental samples [14, 18–20, 22–24]. Therefore, we sought to test the growth dynamics of a non-axenic algal strain and identify the bacteria it harbors. The algal strain *E. huxleyi* CCMP1516, which was isolated from the environment along with an associated bacterial community, was observed to grow in the presence of bacteria without experiencing algal death (Fig. 1A). As a comparison, the axenic isogenic strain *E. huxleyi* CCMP2090, which lacks bacteria, exhibited similar growth in pure culture but experienced algal death when co-cultured with pathogenic *P. inhibens* DSM17395 bacteria (Fig. 1A). Algal death in the co-culture resembled previous reports [20, 23, 24].

To identify the bacteria associated with *E. huxleyi* CCMP1516, a 16S rRNA gene amplicon sequencing analysis was performed at three different time points representing different physiological stages of the alga: mid exponential phase (day 7), late exponential phase (day 10), and early stationary phase (day 14) (Fig. 1A). This analysis revealed that all bacteria in the community are common heterotrophic marine bacteria from the Proteobacteria phylum, belonging to five different taxonomic families including *Alteromonadaceae*, *Marinobacteraceae*, *Rhodobacteraceae*, *Sphingomonadaceae* and *Piscirickettsiaceae* (Fig. 1B). Two OTUs were identified as belonging to the *Rhodobacteraceae* family (Roseobacter group [28]), with one OTU belonging to the *Sulfitobacter* genus and the other not identified at the genus level. The limited diversity observed at the phylum level, as compared to environmental samples [4–7], may be attributed to the historical isolation of the algal strain *E. huxleyi* CCMP1516 several decades ago by the National Center for Marine Algae and Microbiota (NCMA). Consequently, its associated bacterial community likely reflects a population that has been selectively adapted to laboratory culture conditions over the years. However, it is worth noting that the bacterial composition of this algal strain does encompass the two most prevalent classes of bacteria, namely alphaproteobacteria and gammaproteobacteria, found in the deep chlorophyll maxima (DCM) and sea surface layers (SRF) of environmental samples [38]. Additionally, the presence of members of the Roseobacter group within the bacterial population is particularly noteworthy due to their known pathogenic interactions with *E. huxleyi* [14, 18–20, 22–24].

### The pathogenicity of the *P. inhibens* isolate is executed in the absence of the bacterial community

**Fig. 2:**
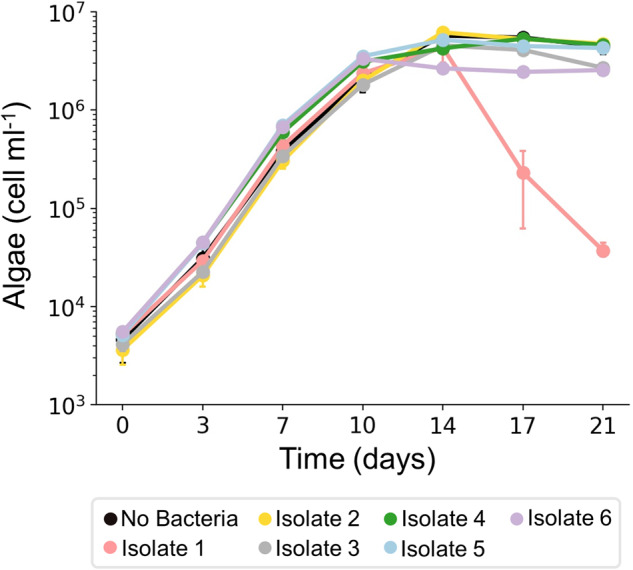
Growth of algae in co-cultures with isolated bacteria. Growth of axenic *E. huxleyi* CCMP2090 was monitored in co-cultures, each time with a different isolate, for 21 days. As can be seen, algal death was triggered only in co-cultures with isolate #1. Each data point consists of 3 biological replicates, error bars designate ± SD. The growth of isolate #1 in the co-culture (*P. inhibens* i.) is presented in Supplementary Fig. S2C.

The algal strain *E. huxleyi* CCMP1516 exhibits uninterrupted growth (Fig. 1A), suggesting that the bacteria associated with it are non-pathogenic. To determine whether indeed bacteria in the community are non-pathogenic, bacteria from *E. huxleyi* CCMP1516 were isolated, and each isolate was co-cultured with the isogenic axenic strain *E. huxleyi* CCMP2090. Six different bacterial isolates were retrieved based on colony morphology (see materials and methods), and their influence on algal growth was monitored along time (Fig. 2). One of these isolates, isolate #1, induced algal death in co-culture (Fig. 2). To identify the pathogenic isolate and the other isolates, a PacBio whole genome sequencing analysis was performed. We found that the 6 isolates belong to the taxonomic families of *Alteromonadaceae*, *Marinobacteraceae*, *Rhodobacteraceae* and *Sphingomonadaceae* (see Table 2 and materials and methods). The isolated bacteria represent four of the five taxonomic families associated with *E. huxleyi* CCMP1516 that we identified earlier using 16S rRNA gene sequencing (Fig. 1B). The bacterial isolates were identified at the species level (see Table 2, materials and methods, and Supplementary Table S1). The pathogenic isolate was identified as a *P. inhibens* strain, a well-studied *E. huxleyi* pathogen [20, 23, 24]. These results reveal that the isolated *P. inhibens* strain does have pathogenic capabilities towards algae in co-culture, however, the pathogenicity appears to be suppressed by the bacterial community in a yet unknown manner. From here on, the identified isolates are denoted by the species name followed by the mark i (for example- *P. inhibens* i.). Experiments involving algae were performed using axenic cultures of strain *E. huxleyi* CCMP2090. To enhance readability, this strain will be referred to as ‘algae’ throughout the manuscript.

### A synthetic community allows the study of community-regulation of pathogenicity

**Fig. 3:**
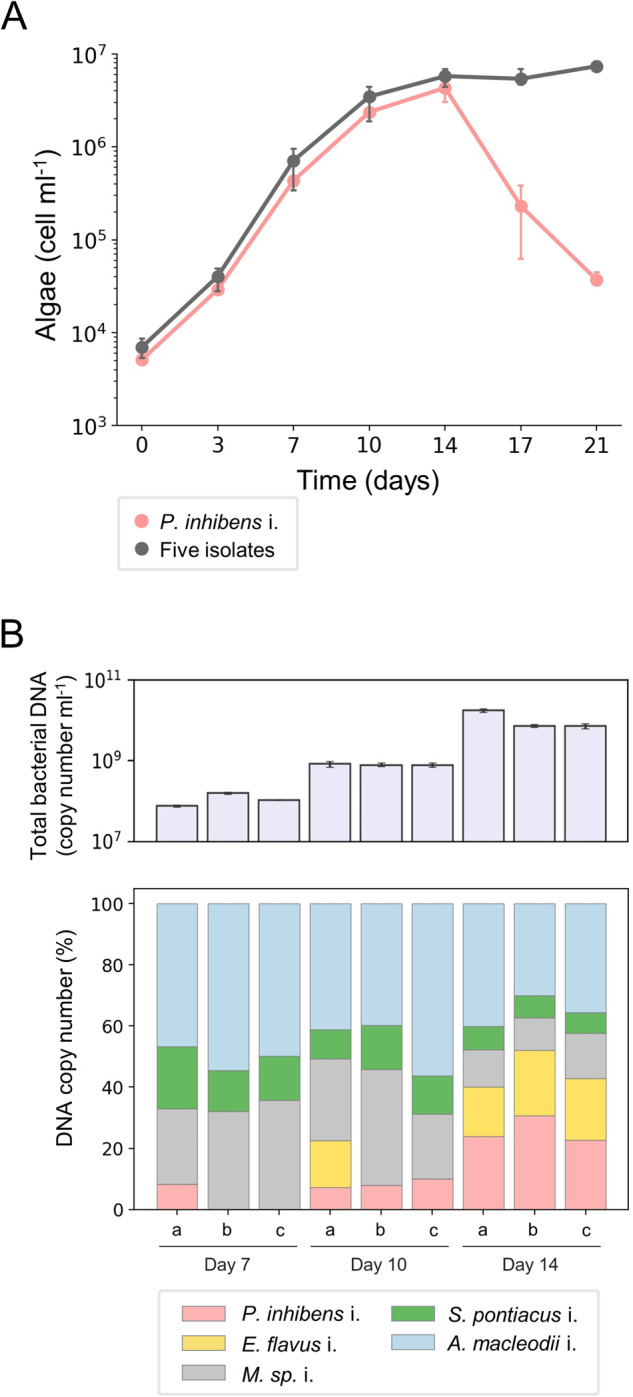
Algae grow in a syncom that contains the isolated bacterial pathogen. **A** Growth of *E. huxleyi* CCMP2090 with *P. inhibens* i. or with the five-member bacterial consortium (syncom) was monitored for 21 days. The data show that algal death is not triggered by the bacterial consortium although it contains the bacterial pathogen. Each data point consists of 3 biological replicates, error bars designate ± SD. The corresponding growth of *P. inhibens* i. in co-culture can be seen in Supplementary Fig. S2C. **B** Upper panel – total bacterial copy number was quantified using a specific DNA locus for each species in the syncom (see materials and methods) along three time points of algal growth. Each time point consists of 3 biological replicates (a, b and c), error bars designate ± SD. Lower panel - the relative abundance of the five isolated bacteria in the syncom was calculated as a percentage of DNA copy number (derived from the total DNA copy number presented in the upper panel) at three time points during algal growth. Of note, the relative abundance of the *P. inhibens* i. appears to increase although pathogenicity is not executed, evident by no algal death. Each time point consists of 3 biological replicates (a, b and c).

To study how a bacterial community regulates the pathogenicity of *P. inhibens* i. towards *E. huxleyi* algae, we aimed to create a synthetic community (syncom) that includes an algal host and the bacterial isolates. This new, reduced and controlled system allowed us to gain insight into the factors that govern the pathogenicity of *P. inhibens* i. in the context of a bacterial community. The syncom was designed to meet two criteria: (I) algae should grow with the bacterial population without induction of algal death, and (II) the pathogenic *P. inhibens* i. should be present and growing in the community over time. Combinations of bacteria were cultured with axenic *E. huxleyi* CCMP2090 until a five-member bacterial consortium was established that met these criteria. The five bacterial species in the syncom included *P. inhibens* i., *Erythrobacter flavus* i., *Marinobacter sp*. i., *Sulfitobacter pontiacus* i. and *Alteromonas macleodii* i. Algae indeed grew over time with the five bacterial isolates without experiencing algal death, in contrast to the abrupt algal death observed in co-cultures with only *P. inhibens* i. (Fig. 3A). To monitor the presence and growth of the different bacteria in the community, particularly *P. inhibens* i., we used a quantitative PCR (qPCR) method that we established for bacteria in the syncom (see the material and methods section for detailed information). The accurate genomes that we sequenced allowed us to identify a unique genomic locus in each isolate and generate specific primers for each species in the community (see Table 3 and material and methods). We note that our qPCR method does not differentiate live and dead bacteria. Yet, calibration experiments with pure bacterial cultures showed good agreement between counts of colony forming units (CFUs) and the qPCR method (Supplementary Fig. S1). Thus, the qPCR method provides a good approximation of total abundance of different bacteria in the syncom. To monitor bacterial growth in the syncom, we chose three time points along the algal growth curve which represent different algal physiological states: mid exponential phase (day 7), late exponential phase (day 10), and early stationary phase (day 14) (Fig. 3A). Results of bacterial qPCR monitoring showed that *P. inhibens* i. was present and growing in the syncom over time (Fig. 3B), reaching its highest density during algal early stationary phase (day 14). The other four isolates were also present in the community and exhibited various growth dynamics over time. Overall, the syncom recapitulated the phenotype observed in the original bacterial population of *E. huxleyi* CCMP1516, where the presence of pathogenic bacteria does not trigger abrupt algal demise. The constrained and regulated composition of the novel algal-bacterial syncom offers an opportunity to explore how the bacterial cohort provides protection to algae against pathogens within the community.

### A single bacterial species in the syncom alleviates the pathogenicity of the *P. inhibens* isolate

**Fig. 4:**
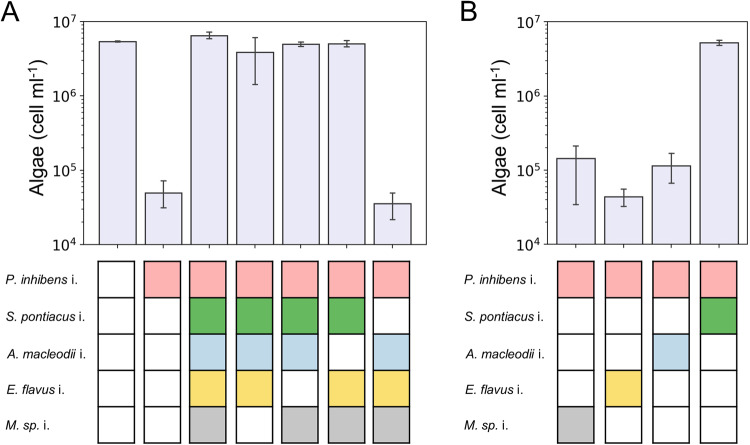
Algal growth with reduced bacterial consortium. **A** *E. huxleyi* CCMP2090 were cultivated with reduced bacterial consortium in which each species was omitted systematically, giving rise to four different 4-member bacterial communities. Shown are algal cell counts on day 21 of growth. In control cultures, algae were cultivated with no bacteria, only with the pathogen *P. inhibens* i., and with the full five-member syncom. The data show that omission of *S. pontiacus* i. from the syncom results in algal death. **B** Algae were cultivated with the pathogen *P. inhibens* i. and with one additional species from the syncom. These cultures of algae with two bacterial species are called tri-cultures. Shown are algal cell counts on day 21 of the tri-cultures. These results show that *S. pontiacus* i. bacteria protect algae from pathogenic *P. inhibens* i. bacteria. The colored rectangles under each bar denote the bacterial species introduced into the algal culture. A white rectangle indicates that the species was not added. Each data point consists of 3 biological replicates, error bars designate ± SD.

The syncom is a reduced and controlled model system in which a bacterial community protects an algal host from a pathogen. To determine whether protection is mediated by the entire community or by a single bacterial species, we systematically omitted from the syncom each bacterial species one at a time, while the pathogen remained present (Fig. 4A). We found that when *S. pontiacus* i. was absent from the syncom, the pathogenic *P. inhibens* i. induced algal death. These observations suggest that a single bacterial species in the community is responsible for algal protection. To further validate these findings, we established tri-cultures in which algae were cultured with the pathogen and one additional bacterial species from the syncom (Fig. 4B). Our data showed that in tri-cultures containing algae with the pathogen and *S. pontiacus* i., no pathogenicity was observed and algae survived. These results demonstrate that a bacterial species belonging to the *Sulfitobacter* genus has the capacity to protect algae from a pathogen.

### Algal protection from bacterial pathogenicity is common in the *Sulfitobacter* genus

**Fig. 5:**
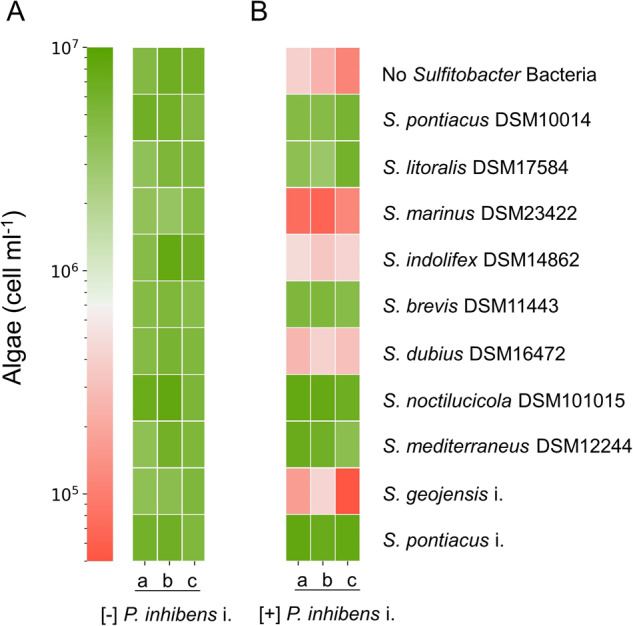
Algal protection in the *Sulfitobacter* genus. **A** *E. huxleyi* CCMP2090 cultured with various *Sulfitobacter* species. **B** *E. huxleyi* CCMP2090 cultured with various *Sulfitobacter* species and with the pathogen *P. inhibens* i. Shown are algal cell counts on day 21 of growth as indicated by the color scale. Each culture was tested using three biological replicates (a, b, c). The corresponding growth of selected *Sulfitobacter* species in co-cultures and tri-cultures can be seen in Supplementary Fig. S2A, B.

We sought to determine the prevalence of the protection capability among the *Sulfitobacter* genus by testing 10 species from this genus. Eight species were obtained from the German Collection of Microorganisms and Cell Cultures (DSMZ), and we isolated two species from *E. huxleyi* CCMP1516 (*S. pontiacus* i. and *S. geojensis* i.). To confirm that these various *Sulfitobacter* species are themselves non-pathogenic towards *E. huxleyi*, we co-cultured each *Sulfitobacter* species with algae (Fig. 5A). None of the *Sulfitobacter* species tested were found to be pathogenic. We then monitored tri-cultures in which algae were cultivated with the pathogenic *P. inhibens* i. and one *Sulfitobacter* species (Fig. 5B). Our results revealed that six *Sulfitobacter* species were able to protect algae from the pathogen, while four species were neutral and did not prevent algal death. Importantly, the cell density of protector or neutral *Sulfitobacter* species was not influenced by the presence of the pathogen (Supplementary Fig. S2A, B). However, the cell density of the pathogen was slightly reduced by both protector and neutral *Sulfitobacter* species regardless of algal fate (Supplementary Fig. S2C). These results suggest that the mechanism of protection is independent from the observed changes in cell density of the pathogen. A phylogenetic tree of the *Sulfitobacter* species was constructed to test whether the protection phenotype has a common evolutionary ancestor (Supplementary Fig. S3). This analysis showed that the protector phenotype is scattered throughout the tree and that protector *Sulfitobacter* species do not have a distinct, close phylogenetic relationship with each other. Next, we tested whether the genomes of the *Sulfitobacter* species could be used to identify functions related to the protection phenotype (Supplementary Fig. S4). We focused on traits that are present in all protector *Sulfitobacter* species but absent from all neutral *Sulfitobacter* species. The functional analysis did not reveal common features that are unique to protector *Sulfitobacter* species. While the mechanism of *Sulfitobacter* protection remains to be determined, this capacity is common among this genus and may therefore present an advantage for harboring *Sulfitobacter* species in the algal microbiome.

### *Sulfitobacter* species are commonly associated with phytoplankton in the environment

To determine whether *Sulfitobacter* species tend to co-occur with microalgae in the environment, we analyzed data from the Tara expedition using the Ocean Barcode Atlas web server [39]. We analyzed the most abundant Roseobacters that are found co-occurring with phytoplankton. Since photosynthesizing microbes inhabit the surface waters and the DCM, we focused our analyses on samples that were retrieved from these water layers and were collected using a small pore size (0.22–3 µm) suitable for capturing bacteria. The data indicated that *Sulfitobacter* species were identified in 34 out of 65 surface samples and in 21 out of 37 DCM samples (Supplementary Fig. S5A), with a relative abundance ranging 0.1–14% of the Roseobacters species in these samples (Supplementary Fig. S5B). These data show that *Sulfitobacter* species commonly occur in waters known to inhabit phytoplankton, yet their average relative abundance is quite low. To determine whether low *Sulfitobacter* abundance confers protection in laboratory settings, we monitored algal cultures in which protector bacteria had an initial abundance ranging from 0.01 to 1% relative to the pathogenic bacteria abundance (Supplementary Fig. S6). Our results showed that protector *Sulfitobacter* bacteria could still protect algae even at extremely low initial abundance compared to the pathogen. These data indicate that the protection capability is robust even when protector bacteria are outnumbered by pathogenic bacteria.

*Sulfitobacter* species are often present in environmental marine samples and algal microbiomes [5, 7, 17, 32, 40–42]. Our data point to the advantageous properties of *Sulfitobacter* species in algal microbiomes where they potentially protect algae from bacterial pathogenicity.

## Discussion

In this study, we present an exploration of algal-bacterial interactions through the use of an environmentally inspired synthetic community (syncom). This approach allowed us to investigate intricate interactions with increased bacterial complexity and to uncover previously unknown ecological roles within these interactions. While simplified algal-bacterial co-cultures are valuable for studying the mechanisms underlying these interactions, the enhanced complexity of syncoms [43] allowed the detection of the novel protector role.

Our results reveal a key bacterial species, *S. pontiacus* i., which protects algae from a pathogen present within the community. Our syncom is environmentally relevant, as the bacteria were isolated from the microbiome of an algal strain (Fig. 1B, Supplementary Table S1). The reduced and controlled nature of the syncom allowed us to systematically eliminate bacteria from the community and identify the protection phenotype in a single bacterial species (Fig. 4A, B). Importantly, due to the high level of genomic diversity in *E. huxleyi* strains [36], our results could represent a strain-specific interaction between algae and bacteria. Notably, previous research has demonstrated that specific characteristics of algal-bacterial interactions play a role in multiple algal-bacterial pairs. For instance, the production of the phytohormone indole-3-acetic acid (IAA) by algal-associated bacteria has been found to impact interactions with various phytoplankton species, including coccolithophores [20], diatoms [17], and chlorophytes [44]. Moreover, the pathogenicity of bacteria towards algae is not exclusively determined by specific algal-bacterial pairs. This has been substantiated by studies illustrating the ability of certain bacterial species to exhibit pathogenic behavior towards various algal strains [20, 22, 45]. Further studies on the protection phenotype using other *E. huxleyi* strains and additional phytoplankton groups will provide insights into the universality of this interaction.

Bacteria protecting their host is a known phenomenon in various natural systems [33, 46, 47]. Our study is the first to show this behavior in algal-bacterial interactions. Mechanisms of host protection by bacteria include competition between bacteria through antibiotic secretion [48], promotion of host defense systems [49], and interference with quorum sensing molecules required for virulence of pathogens [50]. In the current study, both protector and neutral bacteria slightly reduced the cell density of the pathogen (Supplementary Fig. S2C). Therefore, this inhibitory effect cannot explain the protection phenotype. The lack of commonalities in the phylogenetic and functional analyses among protector bacteria (Supplementary Figs. S3 and S4) suggests that the regulation of the protector phenotype could therefore be on a different cellular level (e.g., gene variance, transcription, protein expression). We made initial attempts to elucidate possible *Sulfitobacter* protection mechanisms that interfere with known pathogenicity routes of *P. inhibens* such as secretion of the IAA [20] and nitric oxide [45]. While our initial investigations have not yet revealed the precise mechanism of *Sulfitobacter* protection, ongoing research in our laboratory aims to decipher this complex interplay.

Considering the ubiquity of *Sulfitobacter* species in the marine environment (Supplementary Fig. S5), the observed protection behavior, which was found to be common among the species we tested (Fig. 5), may be of ecological significance. The ability of these bacteria to provide protection even at low initial abundances (Supplementary Fig. S6) raises questions about their role in maintaining algal health and influencing population dynamics. Our study, together with previous findings [22], reveals the multifaceted interactions of *Sulfitobacter* species with the coccolithophore microalga *E. huxleyi*—including pathogenic [22], neutral, and protector roles. This complexity prompts inquires into the factors governing the nature of *Sulfitobacter*-algal interactions and whether the environmental context can modulate these interactions. With advances in *in situ* genetic manipulation techniques [51], hypotheses about the protection mechanism and its environmental significance could be explored.

As our oceans experience global transformations, populations of marine microorganisms shift as well [52]. Perturbations in environmental factors such as salinity [53], pH [54] and temperature [55] could potentially disrupt the balance between pathogens and protectors in natural microbial communities. Controlled laboratory settings provide a platform to investigate the protection phenotype under conditions mimicking anticipated oceanic changes, advancing our understanding of algal-bacterial ecophysiology in changing environments.

## Materials and methods

### Strains and general growth conditions

The algal strains of *E. huxleyi* CCMP1516 (non-axenic) and CCMP2090 (axenic re-isolate of CCMP1516) were purchased from the National Center for Marine Algae and Microbiota (Bigelow Laboratory for Ocean Sciences, Maine, USA). Algae were grown in L1 medium without Na_2_SiO_3_ (L1-Si), based on filtered and autoclaved seawater (FSW) according to Guillard and Hargraves [56]. Algae were grown in standing cultures in a growth room at 18 °C under a light/dark cycle of 16/8 h. Illumination intensity during the light period was 130 μmol photons m^−2^ s^−1^. Absence of bacteria in axenic algal cultures was monitored weekly both by plating on ½ YTSS plates and under the microscope.

The bacterial strains of *Phaeobacter inhibens* DSM 17395, *Sulfitobacter noctilucicola* DSM 101015, *Sulfitobacter indolifex* DSM 14862, *Sulfitobacter dubius* DSM 16472, *Sulfitobacter litoralis* DSM 17584, *Sulfitobacter pontiacus* DSM 10014, *Sulfitobacter marinus* DSM 23422, *Sulfitobacter mediterraneus* DSM 12244 and *Sulfitobacter brevis* DSM 11443 were purchased from the German collection of microorganisms and cell cultures (DSMZ, Braunschweig, Germany). All bacteria were grown in Marine Broth 2216 medium (MB) (Difco, USA) in liquid cultures and agar plates. *Phaeobacter inhibens* DSM 17395 was grown in ½ YTSS medium (2 g yeast extract, 1.25 g tryptone and 20 g sea salt per liter. Sigma-Aldrich, USA) in liquid cultures and agar plates.

### Algal counts

Algal growth in cultures was monitored by a CellStream CS-100496 flow cytometer (Merck, Darmstadt, Germany), using 561 nm excitation and 702 nm emission. For each sample 50,000 events were recorded. Algal cells were gated according to event size and fluorescence intensity.

### Algal culture conditions

*E. huxleyi* was cultured as follows: algal cell concentrations from one week-old *E. huxleyi* culture were determined as described above. An inoculum of 10^4^ algal cells was introduced into 30 ml of L1-Si medium and incubated as described above. In all experiments the bacterial strains were inoculated on the fourth day of algal cultivation and this point was designated as day 0, including control experiments where the alga was cultivated axenically.

Inoculation of bacteria to axenic *E. huxleyi* CCMP2090 was performed as follows: Bacteria were plated from a glycerol stock on either ½ YTSS or MB plates according to Table 1 and incubated at 30 °C for 48 h. Biomass from the plate was then transferred to liquid ½ YTSS or MB and incubated shaking at 30 °C for 48 h. One ml from the liquid bacterial culture were centrifuged at 10,000 rpm for 1 min and washed with FSW three times. The resulting washed pellet was resuspended in 1 ml FSW and diluted to an OD_600_ of 0.01. The diluted sample was then further diluted by a factor indicated in Table 1. From the final dilution, 20 µl were introduced into 30 ml of algal culture grown for four days as described above. Inoculation of multiple bacteria to an algal culture (syncom or tri-culture) followed the same protocol as described above with the addition of a bacterial mixing step: from the final dilution, 20 µl from each bacteria were transferred to an eppendorf tube and mixed. The inoculation volume to the algal culture corresponded to the number of desired bacteria (i.e. if two bacteria are inoculated, 40 µl was transferred to the algal culture). The bacterial starting cell concentration was verified by plating and counting colony forming units (CFUs), resulting in 5-30 CFUs per 20 µl inoculum.

**Table 1:** List of bacteria used in this study and culturing conditions.

	Strain	Origin	Reference genome	Growth medium	Dilution factor
**1**	*Phaeobacter inhibens* DSM 17395	DSMZ, Braunschweig, Germany	GCF_000154765.2	½ YTSS	x10^2^
**2**	*Sulfitobacter noctilucicola* DSM 101015	DSMZ, Braunschweig, Germany	GCF_014197555.1	Marine broth	x10^2^
**3**	*Sulfitobacter indolifex* DSM 14862	DSMZ, Braunschweig, Germany	GCF_022788655.1	Marine broth	x10^3^
**4**	*Sulfitobacter dubius* DSM 16472	DSMZ, Braunschweig, Germany	GCF_900113435.1	Marine broth	x10^3^
**5**	*Sulfitobacter litoralis* DSM 17584	DSMZ, Braunschweig, Germany	GCF_900103185.1	Marine broth	x10^3^
**6**	*Sulfitobacter pontiacus* DSM 10014	DSMZ, Braunschweig, Germany	GCF_900106935.1	½ YTSS	x10^3^
**7**	*Sulfitobacter marinus* DSM 23422	DSMZ, Braunschweig, Germany	GCF_900116285.1	Marine broth	x10^3^
**8**	*Sulfitobacter mediterraneus* DSM 12244	DSMZ, Braunschweig, Germany	GCF_003054325.1	Marine broth	x10^3^
**9**	*Sulfitobacter brevis* DSM 11443	DSMZ, Braunschweig, Germany	GCF_900112755.1	Marine broth	x10^3^
**10**	*Phaeobacter inhibens* i (iR1)	Isolated from *Emiliania huxleyi* CCMP1516	10.5281/zenodo.7520018	½ YTSS	x10^2^
**11**	*Erythrobacter flavus* i (iR2)	Isolated from *Emiliania huxleyi* CCMP1516	10.5281/zenodo.7520027	½ YTSS	x10^3^
**12**	*Marinobacter sp*. i (iR3)	Isolated from *Emiliania huxleyi* CCMP1516	10.5281/zenodo.7520020	½ YTSS	x10^2^
**13**	*Sulfitobacter pontiacus* i (iR4)	Isolated from *Emiliania huxleyi* CCMP1516	10.5281/zenodo.7520022	½ YTSS	x10^3^
**14**	*Alteromonas macleodii* i (iR5)	Isolated from *Emiliania huxleyi* CCMP1516	10.5281/zenodo.7519869	½ YTSS	x10^4^
**15**	*Sulfitobacter geojensis* i (iR6)	Isolated from *Emiliania huxleyi* CCMP1516	10.5281/zenodo.7520006	Marine broth	x10^3^

### 16S rRNA amplicon sequencing and data analysis

*E. huxleyi* CCMP1516 was grown for 14 days as described above. On days 7, 10 and 14 three samples were harvested for genomic DNA extraction. Twenty five ml from the algal culture were centrifuged at 10,000 rpm for 20 min. The algal-bacterial pellet was then subjected to DNA extraction using the Wizard Genomic DNA Purification kit (Promega, Madison, USA) following the manufacturer protocol for bacterial DNA extraction.

Genomic DNA was PCR-amplified with primers CS1_515F and CS2_926R (ACACTGACGACATGGTTCTACAGTGTGYCAGCMGCCGCGGTAA and TACGGTAGCAGAGACTTGGTCTCCGYCAATTYMTTTRAGTTT, respectively. Underlined regions represent linker sequences. Modified from Walters et al. 2015 [57]) targeting the V4-V5 variable regions of the microbial small subunit ribosomal RNA genes. Amplicons were generated using a two-stage PCR amplification protocol as described previously [58]. The primers contained 5’ common sequence tags (known as common sequence 1 and 2, CS1 and CS2). First stage PCR amplifications were performed in 10 µl reactions in 96-well plates, using repliQa HiFi ToughMix (Quantabio, USA). PCR conditions were 98 °C for 2 min, followed by 28 cycles of 98 °C for 10 s, 50 °C for 1 s and 68 °C for 1 s. Subsequently, a second PCR amplification was performed in 10 µl reactions in 96-well plates using repliQa HiFi ToughMix. Each well received a separate primer pair with a unique 10-base barcode, obtained from the Access Array Barcode Library for Illumina (Fluidigm, South San Francisco, CA; Item# 100-4876). One microliter of PCR product from the first stage amplification was used as template for the 2^nd^ stage, without cleanup. Cycling conditions were 98 °C for 2 min, followed by 8 cycles of 98 °C for 10 s, 60 °C for 1 s and 68 °C for 1 s. Libraries were then pooled and sequenced with a 15% phiX spike-in on an Illumina MiSeq sequencer employing V3 chemistry (2×300 base paired-end reads). Library preparation and sequencing were performed at the Genomics and Microbiome Core Facility (GMCF) at Rush University.

Microbiome bioinformatics were performed with QIIME2 2021.11 [59]. Quality of raw sequence data was checked using FastQC and the sequences were merged using PEAR. Adapter sequences were removed using the cutadapt algorithm and reads less than 300 bp were removed. Merged and length-filtered sequences were quality filtered using the q2‐demux plugin followed by denoising with DADA2 (via q2‐dada2) [60]. Taxonomy was assigned to ASVs using the q2‐feature‐classifier [61] taxonomy classifier against the SILVA 138 SSU non-redundant-99% reference database [62]. Reagent microbial contaminants were identified and removed using decontam package based on the prevalence of the ASVs in the reagent negative blank controls using default parameters. Samples were rarefied to a depth of 10,000 sequences/sample for downstream analysis.

### Bacterial isolation

Bacteria were isolated from *E. huxleyi* CCMP1516 as follows: algae were grown as described above. On day 7, 10 and 14 of algal growth a 1 ml sample from the algal culture was serially diluted in FSW. Twenty microliters from each dilution were plated on ½ YTSS and MB agar plates using glass beads. Six single colonies with distinct morphologies were picked and re-streaked on agar plates. Single colony picking and re-streaking was performed sequentially three times. Bacterial biomass was then transferred to 20% glycerol stocks and stored at −80 °C.

### PacBio library preparation, sequencing, read processing and taxonomic classification

For PacBio long-read sequencing, multiplexed microbial libraries were prepared, using SMRTBell Express Prep Kit 2.0 (Pacific Biosciences, Menlo Park, CA). DNA was extracted from the six bacterial isolates using Wizard Genomic DNA Purification kit (Promega, Madison, USA) following the manufacturer protocol for bacterial DNA extraction, resulting in 6 genomic DNA samples. A single PacBio sequencing library was constructed in order to sequence the entire genomes of the bacterial isolates. The library was used for SMRTbell template preparation as described in the protocol (Pacific Biosciences, Menlo Park, CA). The resulting SMRTbell template was annealed to the sequencing polymerase, using a Sequel binding kit 3.0. Sequencing was conducted with a 12pM on-plate concentration using a Sequel I System in CLR mode and a single SMRT Cell (1 M; PacBio). PacBio raw reads (polymerase reads) were demultiplexed and assembled using the SMRT Link software (v8.0).

The phylogenetic classification of all bacterial isolates in the current study was determined using two approaches: 16S rRNA gene sequences were analyzed using the online portal of SILVA SINA database [63], assigning classification at the genus level (Table 2). Entire genomes were analyzed to the species level using the GTDB-Tk v1.7.0 [64] software toolkit for assigning objective taxonomic classifications to bacterial and archaeal genomes (Table 2). The two different approaches yielded identical results, confirming the identity of the isolated bacteria at the species level.

**Table 2:** Taxonomic classification of bacterial isolates using two approaches.

	SILVA SINA (16S rRNA) [58]		GTDB-Tk (whole genome) [59]		Isolate identifier in manuscript
	Classification	Identity^a^ (%)	Classification	Identity^a^ (%)	
**1**	Bacteria;Proteobacteria;Alphaproteobacteria;Rhodobacterales;Rhodobacteraceae;Phaeobacter;	99.85	Bacteria;Proteobacteria;Alphaproteobacteria;Rhodobacterales;Rhodobacteraceae;Phaeobacter;inhibens DSM 16374	97.93	*Phaeobacter inhibens* i.
**2**	Bacteria;Proteobacteria;Alphaproteobacteria;Sphingomonadales;Erythrobacteraceae;Erythrobacter;	100	Bacteria;Proteobacteria;Alphaproteobacteria;Sphingomonadales;Erythrobacteraceae;Erythrobacter;flavus DSM 16421	97.93	*Erytherobacter flavus* i.
**3**	Bacteria;Proteobacteria;Gammaproteobacteria;Pseudomonadales;Marinobacteraceae;Marinobacter;	99.45	Bacteria;Proteobacteria;Gammaproteobacteria;Pseudomonadales;Marinobacteraceae;Marinobacter; sp. EhC06	99.99	*Marinobacter sp.* i.
**4**	Bacteria;Proteobacteria;Alphaproteobacteria;Rhodobacterales;Rhodobacteraceae;Sulfitobacter;	99.92	Bacteria;Proteobacteria;Alphaproteobacteria;Rhodobacterales;Rhodobacteraceae;Sulfitobacter;pontiacus DSM 10014	97.28	*Sulfitobacter pontiacus* i.
**5**	Bacteria;Proteobacteria;Gammaproteobacteria;Enterobacterales;Alteromonadaceae;Alteromonas;	99.79	Bacteria;Proteobacteria;Gammaproteobacteria;Enterobacterales;Alteromonadaceae;Alteromonas;macleodii ATCC 27126	97.86	*Alteromonas macleodii* i.
**6**	Bacteria;Proteobacteria;Alphaproteobacteria;Rhodobacterales;Rhodobacteraceae;Sulfitobacter;	99.49	Bacteria;Proteobacteria;Alphaproteobacteria;Rhodobacterales;Rhodobacteraceae;Sulfitobacter;geojensis MM-124	96.32	*Sulfitobacter geojensis* i.

^a^The sequence identity is computed as the number of shared bases (common base-column pairs) divided by the length of the query sequence.

### Monitoring bacterial growth in co-cultures

Bacterial growth in co-cultures was evaluated by sampling co-cultures at different time points, as indicated above. Samples were serially diluted and plated on ½ YTSS or MB plates, depending on the bacteria. CFUs were counted and the concentration in the sampled culture was calculated.

### Monitoring bacterial growth in the syncom using quantitative PCR (qPCR)

Monitoring of bacteria in the syncom with algae was conducted using a qPCR method based on DNA copy number. A species-specific set of primers was designed for each bacterial species using the structural variant calling tool of PacBio SMRT link software (Table 3).

**Table 3:** Species-specific primer sequences for bacterial identification and bacterial genome length.

Bacterial isolate	Forward unique primer	Reverse unique primer	Genome length (bp)
*Phaeobacter inhibens* i.	GCGGCATGATTACGCAGATG	CGAGGATTTGCAGATTGGGC	4,083,657
*Erythrobacter flavus* i.	GCAGGACAGGCCGTATACAT	CCGATCTTCGCCTTCTCCAA	3,013,997
*Marinobacter sp*. i.	ACTGCTTGTCCATACCCTGC	AGCGGGTCCTGTTCTACACA	4,660,702
*Sulfitobacter pontiacus* i.	GCCATCCGCGATCAAAACAA	TACCGTTCAGCTGCCAGAAG	3,509,446
*Alteromonas macleodii* i.	CGCCCACTAAACGAAAATGGA	TACAAAACCCGCTGTGTGC	4,712,091
*Sulfitobacter geojensis* i.	CCCAGCGCATCAAGTCTGAA	CCTAGCTGCGGCTGTATTTG	4,278,117

PCR analyses validated that the primers do not amplify genomic DNA of *E. huxleyi* CCMP2090 and indeed amplify only genomic DNA of the bacterial species of interest. qPCR was conducted in 384 well plates, using SensiFAST SYBR Lo-ROX Kit (Meridian Bioscience) in a QuantStudio 5 (384-well plate) qPCR cycler (Applied Biosystems, Foster City, CA, USA). Accuracy of primers for determining DNA copy numbers was confirmed in two manners: (1) genomic DNA from the different bacteria was mixed in known DNA concentrations and ratios and served as template for the qPCR protocol. (2) Each bacterial species was grown in pure culture to an OD_600_ of 0.2, the culture was plated on an agar plate for CFU counting and in parallel DNA was extracted from the cultures and served as template for the qPCR protocol (Supplementary Fig. S1). The qPCR program ran according to enzyme requirements for 32 cycles. Results were analyzed using a relative standard curve using the QuantStudio 5 software. Primer efficiencies were determined by qPCR amplification of known DNA concentrations. Only primer pairs with a minimum of 80% efficiency were selected.

The syncom of *E. huxleyi* CCMP2090 with the six bacterial isolates was cultured for 14 days as described above. On days 7, 10 and 14, triplicate samples were harvested for genomic DNA extraction as described above. The DNA from all samples was diluted to 1 ng/µl and served as template for the qPCR reaction as described above. To convert the results from ng DNA to DNA copy number the following formula was used (see also Table 3):

$${DNA}\,{copy}\,{number}=\frac{{DNA}\,{concentration}\times 6.022{\times 10}^{23}}{{Genome}\,{length}{\times 10}^{9}\times 650}$$

6.022 × 10^23^ – Avogadro’s number

10^9^ – Conversion from g to ng

650 – Average weight of a bp (g/mole)

### Monitoring bacterial growth in tri-cultures

Growth monitoring of *P. inhibens* i. and the *Sulfitobacter* species cultured with *E. huxleyi* CCMP2090 in tri-cultures was conducted using selective plates, as follows: both bacteria grow on MB plates, but only *P. inhibens* i. grows on plates that contain sucrose as the sole carbon source (sucrose 5.5 mM, NH_4_Cl 5 mM and NaSO_4_ 33 mM dissolved in 1 L L1-Si medium). Tri-cultures were sampled at different time points, as indicated, and the samples were serially diluted and plated on an MB plate and on a defined sucrose plate. The MB plate was used to count CFUs of both species and the sucrose plate was used to count CFUs of *P. inhibens* i. The *Sulfitobacter* CFUs were calculated by subducting the CFUs on the sucrose-containing plate from the CFUs on the MB plates.

### Phylogenetic analysis of *Sulfitobacter* species

SSU rRNA gene sequences, representing reference *Sulfitobacter* species, were obtained from SILVA [62] and the novel isolates. The above sequences were used as queries in a blastn [65] search against the SILVA 138.1 SSU non-redundant-99% reference database. The seven best matches, that were not the query itself, were retained for each query, and were organized in a single non-redundant fasta file, along with the query sequences. The sequences were aligned using the L-ins-i algorithm, as implemented in MAFFT [66], and the alignments were trimmed with TrimAl [67], to exclude alignment positions containing more than 40% gaps. Regions with missing data at the beginning or the end of the alignment were trimmed manually. The most likely tree was found through a heuristic search starting with a maximum parsimony tree and the GTR + GAMMA model of sequence evolution. Branch support was computed through a rapid bootstrap approach using 100 replicates. The maximum likelihood tree reconstruction was carried out with RAxML 8.2.12 [68]. Three visualization was carried out with ETE3 [69].

### Variable functional categories in *Sulfitobacter* species

To study the functional variation between the *Sulfitobacter* species, we searched for diagnostic orthologous groups. We additionally summarized the KEGG pathways and COG categories, comprising of orthologous groups which were missing from at least one of the genomes. To recover orthologous groups we first obtained the protein fasta files of the publically available genomes (Protector strains: *S. brevis* DSM11443, GCF_900112755.1; *S. litoralis* DSM17584, GCF_900103185.1; *S. mediterraneus* DSM12244, GCF_003054325.1; *S. noctilucicola* DSM101015, GCF_014197555.1; *S. pontiacus* DSM10014, GCF_900106935.1. Neutral strains: *S. dubius* DSM16472, GCF_900113435.1; *S. indolifex* DSM14862, GCF_022788655.1; *S. marinus* DSM23422, GCF_900116285.1) and predicted protein coding genes in the novel isolates genomes of the protector *S. pontiacus* i., 10.5281/zenodo.7520022, and neutral *S. geojensis* i., 10.5281/zenodo.7520006, using Prokka [70]. Orthologous groups were determined with OrthoFinder 2 [71] using the default inflation parameter value (1.5). Functional annotation of the protein sequences was carried out with eggNOG mapper 2 [72] using the eggNOG DB version 5 [73]. Orthologous groups which were missing from at least one of the genomes were listed. For each such orthologous group, each category or pathway was represented by the copy number of that orthologous groups, in each of the samples. Then, the occurrence of each category or pathway was summarized across orthologous groups. The resulting table was log transformed (supplementary file “functional_categories_log_counts.tsv”).

### Environmental occurrence of the *Sulfitobacter* genus

Environmental data was obtained using the Ocean Barcode Atlas (OBA) server [39]. Data was retrieved from sampling points within the DCM and SRF and the filter size was limited to size fraction of 0.22–3 µm. The tool for community ecological analysis was used to search for the taxonomic family “Rhodobacteraceae” in the “Tara Oceans miTag 16S 18S version 2” dataset. Automatically generated datasets of the most abundant “Rhodobacteraceae” groups at the genus level were manually inspected for the presence and abundance of the *Sulfitobacter* genus.

### Supplementary information

Supplemental Material

Functional Categories Log Counts

## Data Availability

Strains used in this study can be found in Table 1. Isolated bacteria are available from the corresponding author on reasonable request. Genome sequences were deposited in zenodo.org, the accession numbers can be found in Table 1. Primers designed for species identification using qPCR are available in Table 3. Functional data used for Supplementary Fig. S4 is available in the supplementary file “functional_categories_log_counts.tsv”. Environmental data used for Supplementary Fig. S5 was retrieved from https://oba.mio.osupytheas.fr/ocean-atlas/ using the “community ecological analysis” function. Default criteria were used on the database “Tara Oceans miTAGs 16S and 18S version 2” to search for *Rhodobacteraceae* taxonomy.
