# Supplementary material for: Abundant Sulfitobacter marine bacteria protect Emiliania huxleyi algae from pathogenic bacteria
Source: ISME Commun. 2023 Sep 22;3:100. doi: 10.1038/s43705-023-00311-y (PMC10517135; doi:10.1038/s43705-023-00311-y)
Supplement: Supplementary file 1 — Supplemental Material [file 43705_2023_311_MOESM1_ESM.docx]

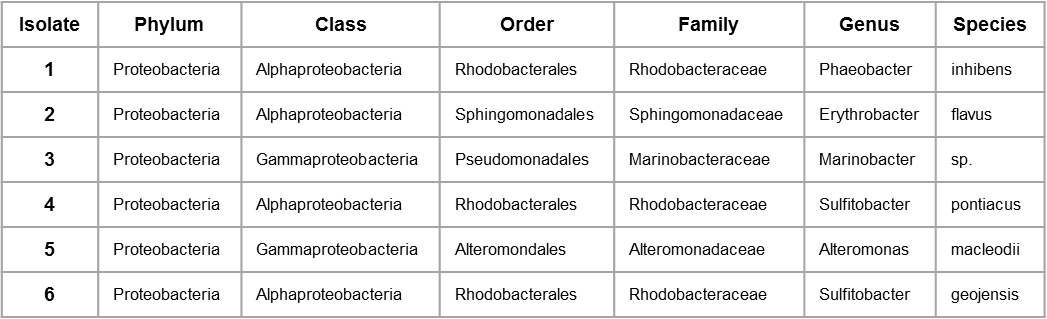


**Table S1. Taxonomy of isolated bacteria.** Six bacteria were isolated from *E. huxleyi* CCMP1516 and sequenced using the PacBio technology to generate full genomes. The taxonomic classification of the bacterial isolates was determined using two independent approaches (see materials and methods). Identified isolates are denoted throughout the manuscript by the species name followed by the mark i (for example- *P. inhibens* i.).


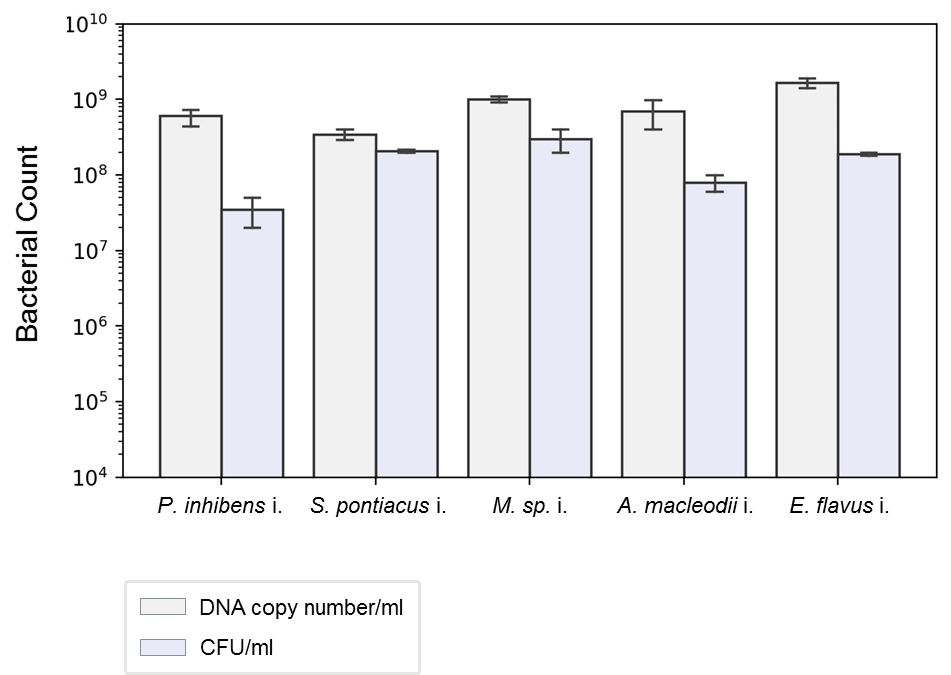


**Fig. S1. Comparison of two methods for bacterial counts.** Bacteria at OD_600_ of 0.2 were either plated on an agar plate for CFU counts or subjected to DNA extraction followed by qPCR for DNA copy number quantification. The data show bacterial counts for each isolate using qPCR (grey bars) and CFU counts (violet bars). Each data point consists of 3 biological replicates, error bars designate ± SD.


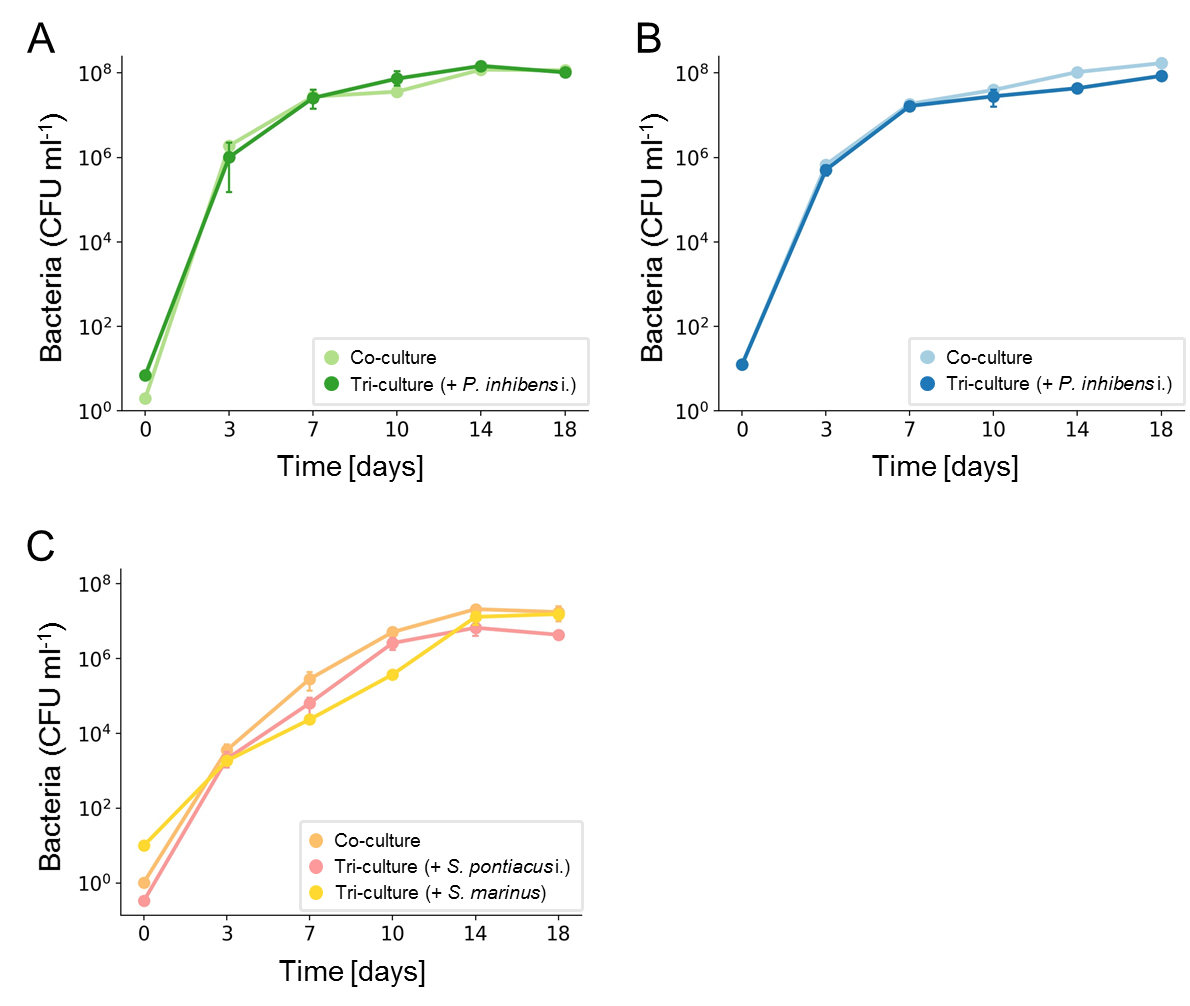


**Fig. S2. Growth of pathogen, protector and neutral bacteria.** (A) Growth of neutral bacteria *S. marinus* DSM23422 was monitored in co-culture with algae, and in tri-culture with algae and the pathogen *P. inhibens* i. along 18 days. (B) Growth of protector bacteria *S. pontiacus* i. was monitored in co-culture with algae, and in tri-culture with algae and the pathogen *P. inhibens* i., along 18 days. (C) Growth of pathogenic bacteria *P. inhibens* i. was monitored in co-culture with algae, in tri-culture with algae and the protector bacteria *S. pontiacus* i., and in in tri-culture with algae and the neutral bacteria *S. marinus* DSM23422 along 18 days. Each data point consists of 3 biological replicates, error bars designate ± SD.


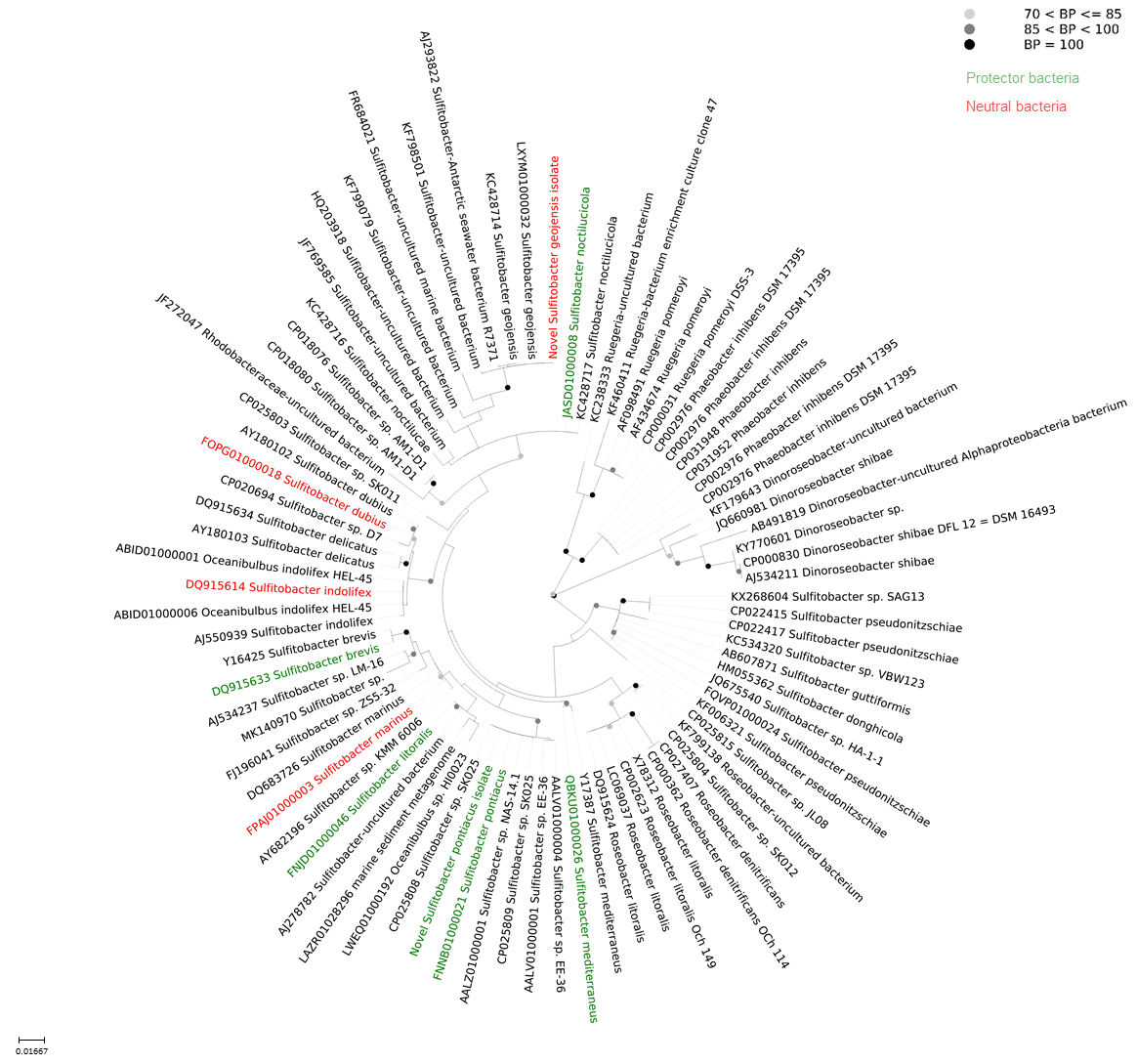


**Fig. S3. Phylogenetic tree of protector and neutral *Sulfitobacter* species within the Roseobacter group.** A 16S rRNA gene sequence maximum likelihood phylogenetic tree of *Sulfitobacter* spp. and closely related species. Node supports are bootstrap percentages (BP) of 100 replications. Light gray bullets at the base of nodes represent 70 < BP <= 85, dark gray bullets denote 85 < BP < 100, and black bullets denote maximal BP support. Leaf labels representing protector and neutral strains are highlighted in green and red respectively. The scale represents a patristic distance of 0.0166 substitutions per base.


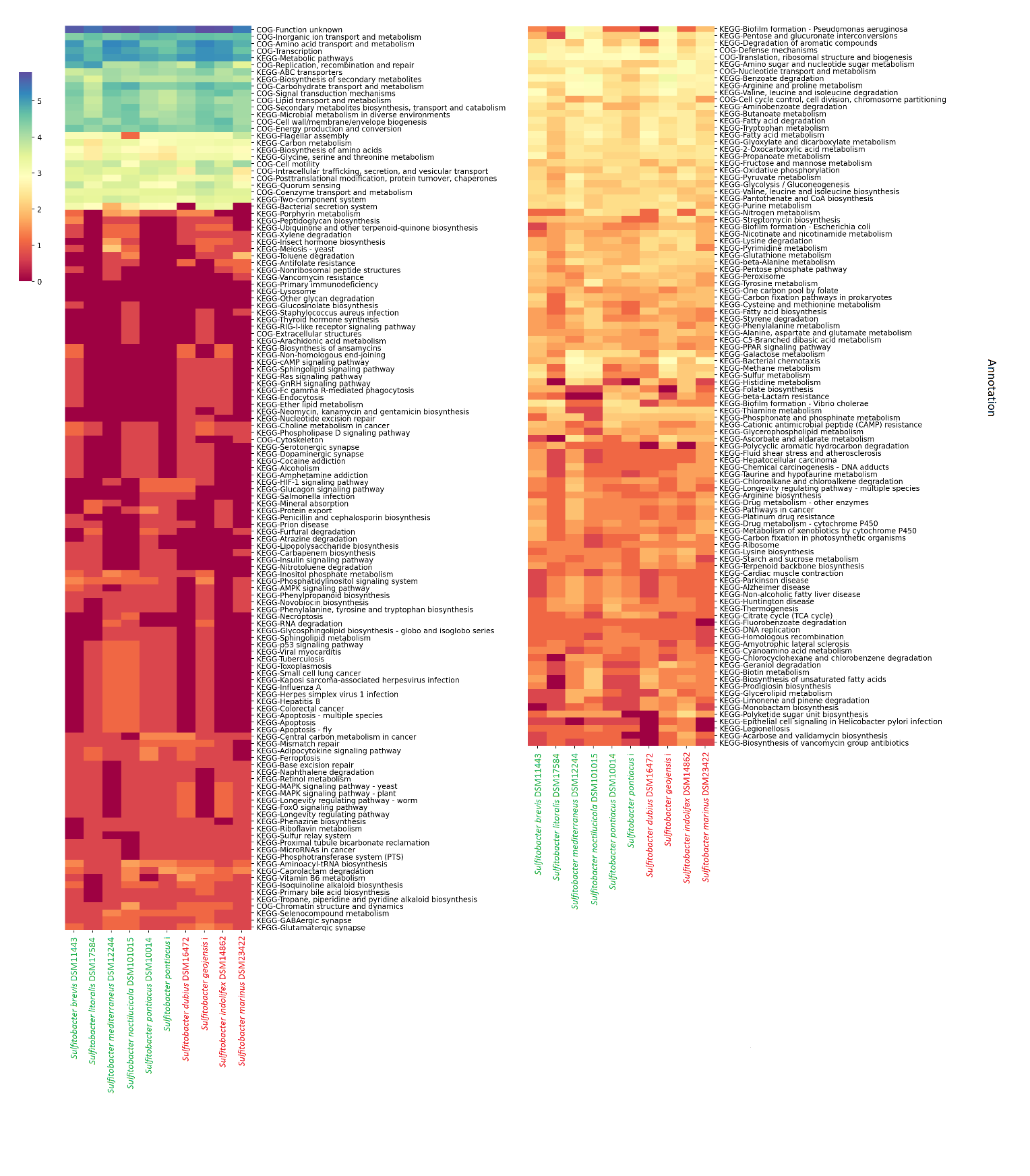


**Fig. S4. Variation in COG functional categories and KEGG pathways in *Sulfitobacter* species.** The x and y axes in the heatmap represent the *Sulfitobacter* species and functional categories, respectively. The color scale represents the number of orthologous groups assigned to each category, multiplied by the copy number of each ortholog in each genome, and log transformed. Colored in green are protector *Sulfitobacter* species, colored in red are neutral *Sulfitobacter* species.


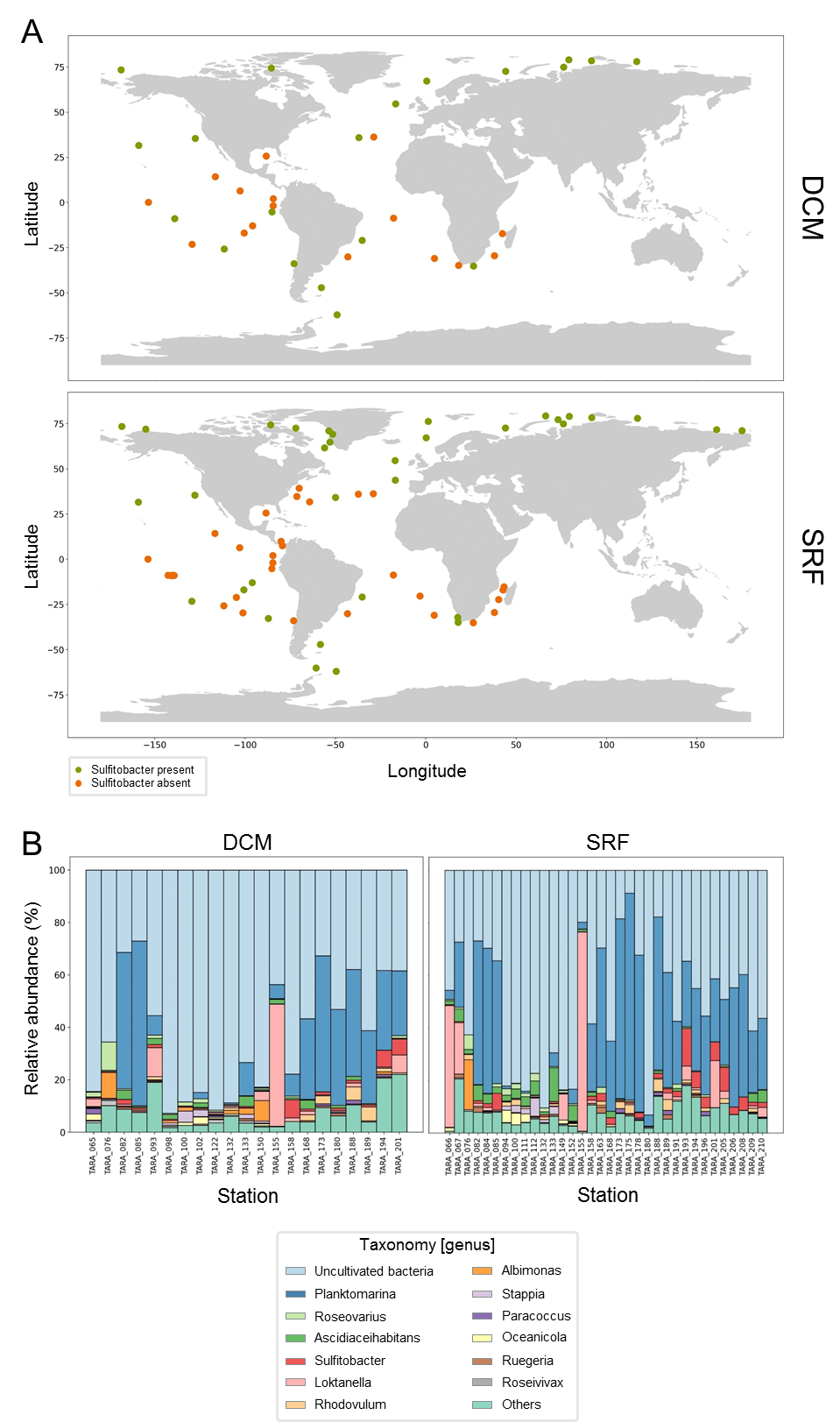


**Fig. S5. Bacteria from the *Sulfitobacter* genus in environmental samples.** Data was generated from the web server Ocean Barcode Atlas. The web server uses taxonomic data from the Tara expedition, according to each station that was sampled in the research. (A) The world maps show the stations that were sampled in the Tara expedition according to the presence (green bullets) or absence (orange bullets) of the *Sulfitobacter* genus. Upper map - deep chlorophyll maximum layer (DCM), lower map - surface layer (SRF). (B) The data show the relative abundance of the 9 most abundant Roseobacters in stations where a Sulfitobacter species was present (corresponding to the maps in panel A). Other Roseobacters are grouped and classified as “others”. Left bar chart – DCM layer, right bar chart – SRF layer. Samples were collected on a 0.22-3µm filter.


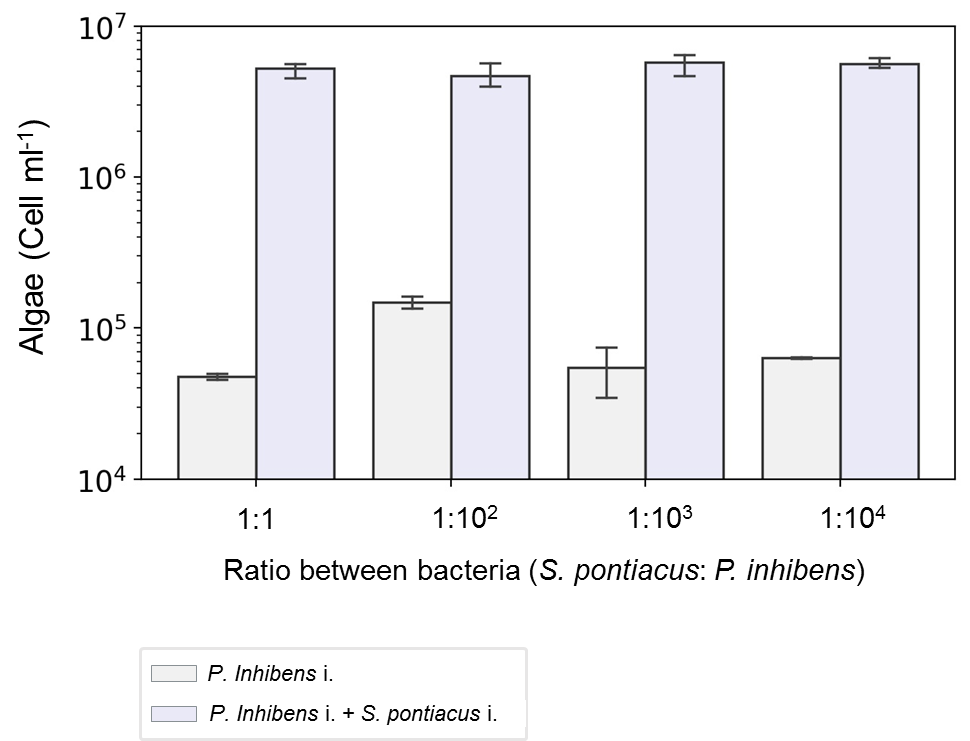


**Fig. S6. Influence of the initial bacterial inoculum on protection capabilities.** Algal cell count on day 21 of growth, cultured with the pathogen *P. inhibens* i. (grey bars) or with the pathogen and the protector *S. pontiacus* i. (violet bars). The initial ratio of the bacterial inoculum is indicated under the bars. Protector bacteria were inoculated in all cases in the same concentration (10 CFU/ml), while the pathogen was inoculated in different and increasing concentrations (10, 10^3^, 10^4^, 10^5^ CFU/ml). Each data point consists of 3 biological replicates, error bars designate ± SD
